# Sex Patterns of Statin Therapy and Multicomponent Exercise Training on Cardiorespiratory Fitness in Older Adults with Dyslipidemia: A 24-Month Cohort Study

**DOI:** 10.3390/sports14060215

**Published:** 2026-05-22

**Authors:** Liliana C. Baptista, Aristides M. Machado-Rodrigues, Marco Antônio Rabelo Da Silva, Elias De França, Raul A. Martins

**Affiliations:** 1 University of Coimbra, Faculty of Sports Sciences and Physical Education, 3040-248 Coimbra, Portugal; a.machado-rodrigues@fcdef.uc.pt (A.M.M.-R.); raulmartins@fcdef.uc.pt (R.A.M.); 2 University of Coimbra, Interdisciplinary Center for the Study of Human Performance, Faculty of Sport Sciences and Physical Education, 3040-248 Coimbra, Portugal; 3 Department of Physical Education, Universidade da Amazónia, Belém 66060-902, Brazil; marcorabelosilva@outlook.com; 4 Interdisciplinary Postgraduate Program in Health Sciences, Universidade Federal de São Paulo, São Paulo 04024-002, Brazil; elias.de.f@hotmail.com

**Keywords:** statins, exercise, sex differences, exercise capacity, older adults

## Abstract

Statins’ effects on cardiorespiratory fitness (CRF) and their interaction with exercise training remain unclear in older adults with dyslipidemia. This cohort study enrolled nine hundred and eighty-one older adults with dyslipidemia who underwent one of three interventions: (i) multicomponent exercise training (MEX; n = 298; 74% females), (ii) daily statin monotherapy (ST; n = 178; 65% females), or (iii) combined treatment with statins and multicomponent exercise training (STMEX; n = 505; 79% females). CRF, functional status, and lipid profile were assessed at baseline and after 24 months. After follow-up, statin therapy reduced CRF by 4% in women (*p* < 0.001), but not in men. The statin groups also showed reduced upper- and lower-limb strength in both sexes. Exercise alone significantly improved CRF (women: 27% vs. men: 21%, *p* < 0.001) and functional status, regardless of sex. The combined treatment significantly increased women’s CRF, whereas men showed an attenuated CRF benefit (women: 27% vs. men: 1%, *p* < 0.001). Our findings suggest sex-specific patterns in the effects of statin therapy on CRF in older adults with dyslipidemia. Statin therapy was associated with lower CRF over time in women, but not in men, whereas multicomponent exercise training may reverse these effects.

## 1. Introduction

Cardiorespiratory fitness (CRF) is a strong predictor of cardiovascular risk and overall mortality, regardless of age, race, and sex [1,2,3]. Increases of just 1 MET (Metabolic equivalent) in CRF are associated with reductions of 13% and 15% in all-cause and cardiovascular mortality risk, respectively [4], whereas low CRF (i.e., <5 METs) is associated with a fourfold higher mortality risk than that of age- and sex-matched fitter counterparts [1]. Several sex-related differences, such as body composition, skeletal muscle characteristics, and cardiorespiratory structure, can significantly influence exercise capacity and contribute to differences in CRF responses to exercise training between men and women [5,6]. These physiological differences are also affected by sex-specific hormonal and metabolic changes that occur with aging [6,7]. Most current evidence [3,8,9] evaluating CRF adaptations to exercise has focused primarily on men, providing little to no attention to responses in older women and how they are affected by aging or pharmacological interventions.

3-Hydroxy-3-methylglutaryl-coenzyme A reductase (HMG-CoA reductase) inhibitors, commonly known as statins, are prescribed to decrease low-density lipoprotein cholesterol (LDL-C) levels [10,11] by competitively inhibiting the active site of the rate-limiting enzyme HMG-CoA reductase, thereby preventing cholesterol biosynthesis and reducing cardiovascular risk [10,12]. Despite their effectiveness, compelling evidence [10,13,14] suggests that statin therapy may induce metabolic and skeletal muscle adverse effects, either alone or in combination with exercise. For instance, statins have been associated with myotoxicity and musculoskeletal conditions ranging from benign myalgia to rhabdomyolysis [10,13,14]. These adverse effects appear to be more common with higher statin dosages, the use of lipophilic statins [15,16], higher levels of exercise training [17], and advanced age [15].

To date, studies investigating the effects of statin treatment on CRF improvements following exercise have reported mixed findings. Several epidemiological and experimental studies [18,19,20,21] have shown that statins do not affect CRF or adaptations in exercise capacity, whereas a few randomized controlled trials (RCTs) have reported that statins may impair CRF and skeletal muscle function after exercise training [22,23]. Notably, studies evaluating CRF following exercise training in older adults [20,24] have not compared sex-specific responses to statin therapy.

Due to the paucity of current evidence, the present study aimed to compare 24-month changes in CRF among inactive older men and women (>60 years) with dyslipidemia undergoing one of three first-line dyslipidemia treatments: (i) multicomponent exercise training (MEX), (ii) statin monotherapy (ST), or (iii) combined statin plus exercise therapy (STMEX). Sex-specific functional performance and blood lipid profiles were also analyzed. We hypothesized that: (a) combined therapy (STMEX) would provide greater benefits than either intervention alone within each sex because of their potential interaction effect [11], as previously suggested [25]; and (b) men would demonstrate greater exercise-induced CRF and functional adaptations than women after the 24-month follow-up.

## 2. Materials and Methods

### 2.1. Study Design Overview

This study is part of a larger prospective non-randomized cohort study focused on analyzing the effects of long-term exercise training, functional status, and other associated cardiovascular risk factors in community-dwelling older adults. The complete methodological design was previously published [25] and is fully described in Supplementary Materials. Briefly, this study involved older adults aged 60 years and older. Follow-up assessment visits occurred at 6, 12, and 24 months using the same baseline methodological testing order. The Institutional Scientific Committee of the University of Coimbra, the local institution Santa Maria da Feira, and national ethics committees, including the Portuguese Data Protection Authority and the North Health Administration Ethics Committee, approved the methods and procedures used in this study. All participants provided written informed consent in accordance with the ethical principles of the 1964 Helsinki Declaration and its later amendments for human research established by the World Medical Association [26].

### 2.2. Participants

This study included participants who: (i) were aged ≥ 60 years; (ii) met the criteria for dyslipidemia established by the European Society of Cardiology and the European Atherosclerosis Society [10]; and (iii) were inactive and physically independent, as determined by responses to the 12-item Composite Physical Functioning Scale [27]. In contrast, participants were excluded if they presented: (a) severe or uncontrolled cardiac conditions; or (b) conditions that made participation in the exercise intervention or attendance at follow-up visits impossible or highly problematic. Participants were also excluded if they: (1) did not complete baseline and follow-up testing; (2) used bile acid sequestrants, cholesterol absorption inhibitors, PCSK9 inhibitors, nicotinic acid, or other drug combinations; or (3) had an exercise training program adherence rate ≤ 80%.

A total of 981 inactive, community-dwelling older adults with dyslipidemia fulfilled all inclusion criteria. Participants were then divided according to three first-line dyslipidemia treatment regimens: (1) multicomponent exercise training only (MEX; n = 298; 74% women); (2) statin monotherapy only (ST; n = 178; 65% women); and (3) combined treatment with statins and multicomponent exercise training (STMEX; n = 505; 79% women).

### 2.3. Intervention

#### 2.3.1. Pharmacological Treatment

Participants in the ST and STMEX groups used daily statin monotherapy prescribed by their primary care physicians according to their lipid profiles for at least one year before study initiation.

#### 2.3.2. Multicomponent Exercise Training

The supervised multicomponent exercise training program (3 sessions/week, 60 min/day) included aerobic, resistance, balance, and flexibility components as follows: a 5–10-min (min) warm-up, 20–30 min of aerobic exercise, 15–20 min of resistance training, 10 min of balance training, 10 min of stretching, and a 5–10-min cool-down. Aerobic exercise consisted of continuous movements involving major upper- and lower-extremity muscle groups performed alternately. The duration and intensity of aerobic exercise progressively increased from 20 to 30 min at 50–70% of maximum heart rate (HRmax) per session [28]. Resistance training involved 5–8 exercises targeting major upper- and lower-body muscle groups, with 1–3 sets of 8–12 repetitions performed using body weight or free weights. Exercise intensity was set at 50–70% of one-repetition maximum (1-RM), with 90–120 s of rest between sets. Balance training involved functional tasks commonly required by older adults. Finally, before the cool-down period, participants performed passive and active stretching exercises (held for 15–30 s, 3 repetitions per exercise) to improve the flexibility of major muscle groups.

This program was designed to meet the exercise and physical activity recommendations for older adults according to the guidelines of the American College of Sports Medicine [28].

### 2.4. Outcomes

The primary aim of this study was to compare longitudinal changes in CRF among community-dwelling older adults with dyslipidemia between sexes. The secondary outcomes included changes in functional status and lipid profiles.

#### 2.4.1. Cardiorespiratory Fitness

CRF was evaluated using the 6-min walk test as a functional proxy within a combined test battery—the Senior Fitness Test battery (validity and test–retest reliability ranging from 0.80 to 0.98) [27]. While the 6-min walk test is a proxy measure rather than a direct assessment of maximal aerobic capacity, the literature has extensively demonstrated its predictive capacity for CRF, exercise capacity, health-related outcomes, and inverse associations with subclinical markers (e.g., inflammation, oxidative stress, and mortality) [29].

#### 2.4.2. Functional Status

The functional status was evaluated using the Senior Fitness Test battery [27], a set of objective tests expressed on a continuous quantitative scale that allows the assessment of longitudinal changes over time (improvements or decline). This battery includes measures of: (1) upper-limb strength (ULS); (2) lower-limb strength (LLS); and (3) velocity, agility, and dynamic balance (VAB). Participants were evaluated between 8:00 and 10:00 a.m.

#### 2.4.3. Lipid Profile

Trained nurses collected venous blood samples in the morning after a 12-h fasting period. Samples were analyzed for glycaemic and lipid profiles. Total cholesterol (TC), high-density lipoprotein cholesterol (HDL-C), low-density lipoprotein cholesterol (LDL-C), triglycerides (TG), glucose, and glycated hemoglobin (HbA1c) were determined using standard methods by the same accredited laboratory throughout the study.

#### 2.4.4. Clinical and Medication History

Participants’ clinical and medication history data were collected using a health questionnaire and through visual confirmation of prescribed medications recorded by trained nurses. The health questionnaire included information on age, sex, education level, living situation, smoking status, and the presence of several conditions, such as heart disease, arterial hypertension, stroke, diabetes, and dyslipidemia.

Trained nurses also measured resting blood pressure, stature, body mass (BM), waist circumference (WC), and hip circumference (details are described in Supplementary Materials). Composite measures, such as body mass index (BMI) and waist-to-hip ratio (WHR), were calculated according to standard methods [30].

### 2.5. Statistical Analysis

The baseline demographic and clinical characteristics were compared within each sex and across the three treatment groups using measures of frequency, central tendency, and dispersion, and are expressed as mean ± standard deviation (SD). One-way analysis of variance (ANOVA), followed by Gabriel’s post hoc multiple comparison test, was used to assess differences between groups in primary and secondary outcomes.

Longitudinal sex-specific changes across the three treatment groups were evaluated using mixed-model repeated-measures analysis of variance to examine differences from baseline to the 24-month follow-up period. Longitudinal between-group differences after the 24-month follow-up were also assessed using analysis of covariance (ANCOVA), adjusting for age, number of comorbidities, body mass index, systolic and diastolic blood pressure, total cholesterol, and glycaemia. In addition, analyses were adjusted for baseline mean scores to minimize the possibility of reverse causation.

The magnitude of longitudinal changes within each sex and treatment group was calculated using Hedges’ g standardized effect size [31]. Effect sizes were classified as small (<0.20), moderate (0.20–0.79), or large (>0.80) [32]. Finally, percentage change (Δ%) was calculated using the formula [(post − pre)/pre × 100] to determine within-group differences across all variables from baseline to the 24-month assessment.

Data analysis was performed using the Statistical Package for the Social Sciences (SPSS) for Windows (IBM Corp., Chicago, IL, USA), version 26. Statistical tests were two-tailed, and significance was set at 5%. GraphPad Prism (version 9.5.2) was used to visually display the results of the analyses.

## 3. Results

### 3.1. Participant Baseline Characteristics

Baseline demographic, clinical, and hemodynamic characteristics by sex and treatment group are presented in Table 1. The 981 community-dwelling older adults with dyslipidemia were predominantly women (78%) and had a mean (±SD) age of 67 (±7.8) years, body mass index (BMI) of 28.9 (±4.1) kg/m^2^, total cholesterol (TC) of 202 (±39) mg/dL, LDL-C of 124 (±34) mg/dL, and an average of 2.4 (±1.6) comorbidities. The most prevalent comorbidities were hypertension (men: n = 119 [44.4%]; women: n = 339 [47.2%]), osteoarthritis (men: n = 39 [14.6%]; women: n = 245 [31.4%]), osteoporosis (men: n = 11 [4.1%]; women: n = 222 [28.4%]), and type 2 diabetes (men: n = 72 [26.9%]; women: n = 127 [16.3%]). There were no significant differences in comorbidity prevalence between men and women, except for type 2 diabetes, which was more prevalent in women in the combined treatment group (*p* < 0.001).

Among men, 48% used simvastatin, 27% atorvastatin, 15% pravastatin, 6% pitavastatin, and 4% rosuvastatin. Similarly, among women, 50% used simvastatin, 24% atorvastatin, 12% pravastatin, 8% rosuvastatin, and 6% pitavastatin. Doses were maintained throughout the follow-up period.

**Figure 1:**
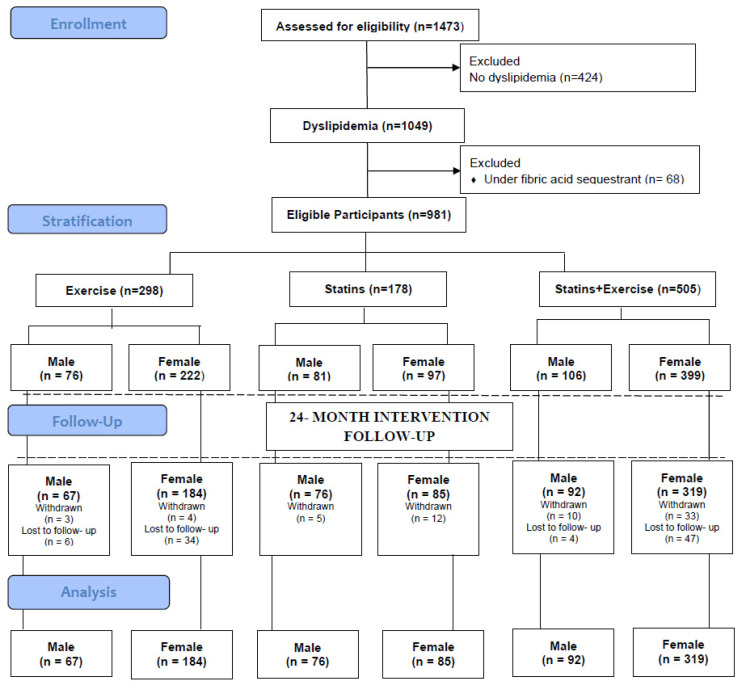
Participants flow throughout the study.

During the 24-month follow-up, 11% of men (n = 28) and 17% of women (n = 130) withdrew or were lost to follow-up (Figure 1). The study was completed by 89% of men (MEX: n = 67 [88%]; ST: n = 76 [94%]; STMEX: n = 92 [87%]) and 83% of women (MEX: n = 184 [83%]; ST: n = 85 [88%]; STMEX: n = 319 [80%]). No serious adverse events occurred during follow-up. Adherence to the exercise intervention was >80% in both sexes (men: MEX = 86%, STMEX = 85%; women: MEX = 82%, STMEX = 80%).

At baseline, there were some significant differences between sexes. Men in the MEX group had fewer comorbidities and lower BMI, WC, and WHR, but higher TC and LDL-C. Men in the ST group were younger, had more comorbidities, and lower SBP and DBP, whereas men in the STMEX group were older and had higher SBP, DBP, BMI, WC, and WHR, but lower TC and LDL-C compared with the other groups. Women in the MEX group had lower fasting glucose and WHR. In contrast, women in the ST group were younger and had lower SBP, DBP, and WC. Women in the STMEX group were older and had higher SBP, DBP, triglycerides (TG), WC, and WHR, but lower LDL-C. There were no significant differences in CRF between sexes or treatment groups at baseline.

### 3.2. Sex Patterns in Cardiorespiratory Fitness

**Figure 2:**
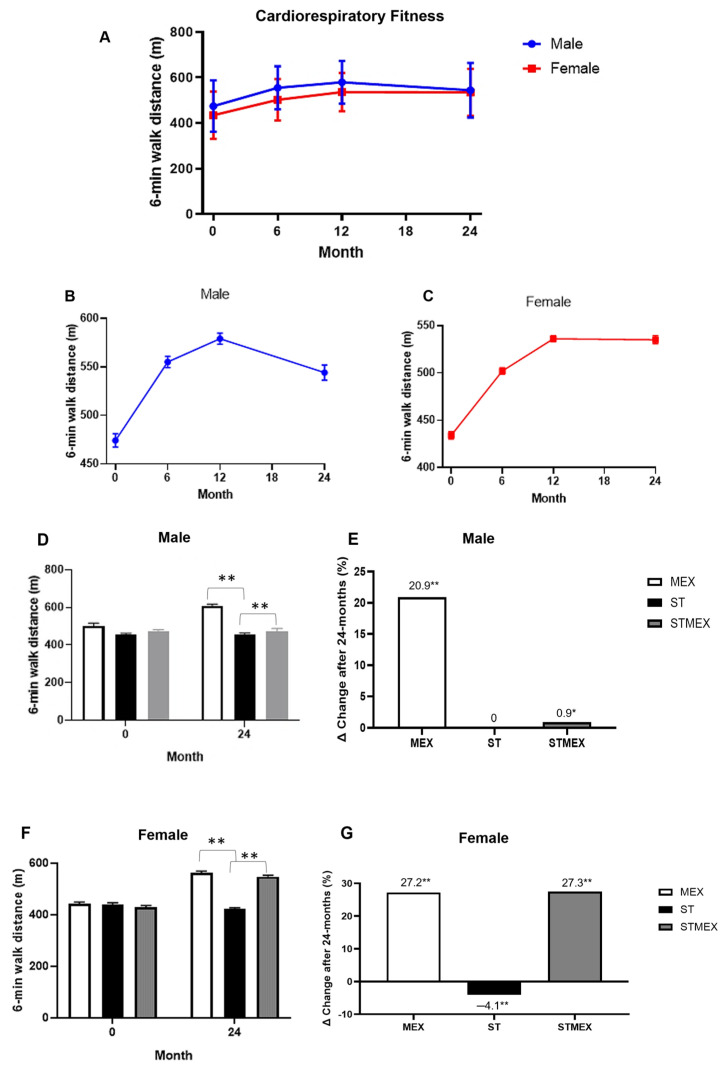
Longitudinal sex-specific changes in cardiorespiratory fitness in older adults with dyslipidemia. After the 24-month follow-up, the combined data showed sex-specific changes (Panels (**A**–**C**)) and within each treatment group. Males in the multicomponent exercise training group (i.e., MEX) improved cardiorespiratory fitness. In contrast, males in the combined treatment group modestly increased the 6-min’ walk distance after the 24-month follow-up compared to males under statin therapy only (Panels (**D**,**E**)). Likewise, females in the MEX and the combined treatment (i.e., STMEX) groups significantly improved cardiorespiratory fitness compared to females under statin therapy (Panels (**F**,**G**)). The longitudinal changes between sexes and within each treatment group were calculated using a repeated measures mixed model analysis followed by a Gabriel post hoc multiple comparison correction test, adjusted to age and comorbidity number. Data are expressed as group means ± SD (N = 981 dyslipidemic community-dwelling older adults). * Statistical significance *p* ≤ 0.05. ** Statistical significance *p* ≤ 0.001.

After the 24-month intervention, there were significant differences between sexes and the three treatment groups in CRF, as shown in Figure 2. Men in the MEX group significantly increased their 6-min walk distance (pre: 501 ± 130 m; post: 606 ± 98 m; *p* < 0.001), with large effect sizes compared with the ST group (Figure 2B,D,E; Supplementary Tables S1–S3). Similarly, men in the STMEX group improved CRF, but to a smaller extent than the MEX group (pre: 471 ± 113 m; post: 475 ± 119 m; *p* < 0.001). Men in the ST group did not show any significant change in CRF over the 24-month follow-up.

Regarding women, a significant treatment effect on CRF was also observed in the MEX group (pre: 441 ± 108 m; post: 561 ± 101 m; *p* < 0.001), with a large effect size, as well as in the STMEX group (pre: 429 ± 110 m; post: 547 ± 97 m; *p* < 0.001; Figure 2C,F,G). Women in the ST group exhibited a small decrease in CRF after the 24-month follow-up (pre: 439 ± 67 m; post: 421 ± 52 m; *p* < 0.001).

### 3.3. Distinct Longitudinal Treatment Changes in Functional Status

**Figure 3:**
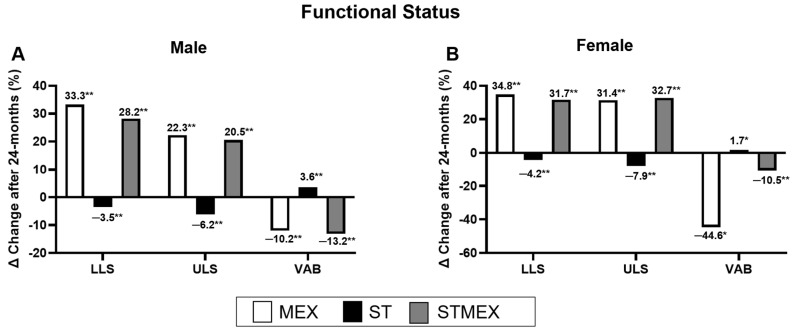
Longitudinal sex-specific changes within each treatment group on functional status in older adults with dyslipidemia. There were sex-specific changes within each treatment group after the 24-month follow-up period. Males in the exercise training groups (i.e., MEX and STMEX) improved upper- and lower-limb strength, and the velocity and dynamic balance, in contrast with males under statin therapy only, which decreased these functional status outcomes (Panel (**A**)). Similarly, females in the MEX and the STMEX groups significantly increased functional status outcomes. In contrast, females under statin therapy decreased upper- and lower-limb strength and worsened their velocity and dynamic balance time after the 24-month follow-up period (Panel (**B**)). The longitudinal changes within gender and each treatment group were calculated using a repeated measures mixed model analysis followed by Gabriel post hoc multiple comparison correction, adjusted to age, comorbidity number and baseline value. Data are expressed as group means ± SD; (Male: n = 235; Female: n = 588). Note: LLS: Lower-limb strength; ULS: Upper-limb strength; VAB: Velocity and dynamic balance. * Statistical significance *p* ≤ 0.05. ** Statistical significance *p* ≤ 0.001.

Men and women in each exercise treatment group exhibited similar longitudinal patterns in functional status outcomes (Figure 3A,B). Men in the exercise groups (i.e., MEX and STMEX) improved upper-limb strength (MEX: 22%; STMEX: 21%; *p* < 0.001), lower-limb strength (MEX: 33%; STMEX: 28%; *p* < 0.001), and velocity, agility, and dynamic balance (MEX: −10%; STMEX: −13%; *p* < 0.001), with large effect sizes (>1.000). In contrast, men in the ST group showed significant reductions in upper- and lower-limb strength (−6% and −4%, respectively; *p* < 0.001) after the 24-month follow-up.

Similarly, women in the MEX and STMEX groups improved across all functional status outcomes, including upper-limb strength (MEX: 31%; STMEX: 33%; *p* < 0.001), lower-limb strength (MEX: 35%; STMEX: 32%; *p* < 0.001), and velocity, agility, and dynamic balance (MEX: −45%; STMEX: −11%; *p* < 0.001), with large effect sizes. Conversely, women in the ST group showed worse functional status outcomes (ULS: −8%; LLS: −4%; and VAB: 2%; *p* < 0.001).

### 3.4. Different Effects of Sex and Treatment on Lipid Metabolic Profile

**Figure 4:**
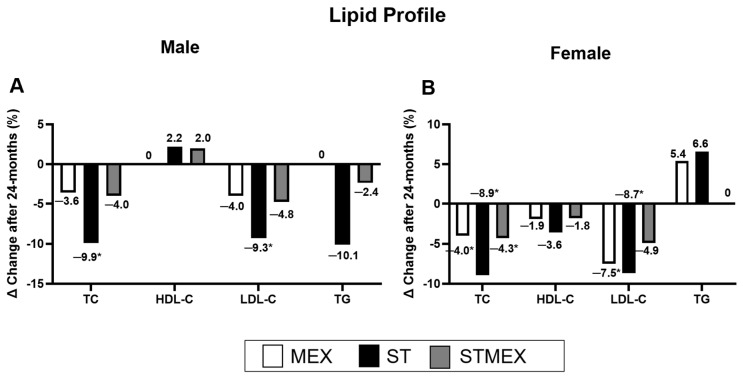
Longitudinal sex-specific changes within each treatment group in lipid profile in older adults with dyslipidemia. After the 24-month follow-up period, males and females in all treatment groups significantly decreased total cholesterol and low-density lipoprotein cholesterol. Males in the statin therapy group had the highest percent change in total cholesterol and LDL-C. In contrast, males in the MEX group were the only group to reduce triglycerides after the 24-month follow-up period (Panel (**A**)). Likewise, females under statin therapy had the highest percent change in total cholesterol and LDL-C, but increased triglycerides after the 24-month follow-up period (Panel (**B**)). The longitudinal changes within gender and each treatment group were calculated using a repeated measures mixed model analysis followed by Gabriel post hoc multiple comparison correction, adjusted to age, comorbidity number and baseline value. Data are expressed as group means ± SD; (Male: n = 235; Female: n = 588). Note: HDL-C: High-density lipoprotein cholesterol; LDL-C: Low-density lipoprotein cholesterol; TC: Total cholesterol; TG: Triglycerides. * Statistical significance *p* ≤ 0.05.

All treatment groups reduced TC and LDL-C after the 24-month intervention, independently of sex (Figure 4A,B). However, both men and women in the ST group showed the greatest reductions in TC (men: −10%; women: −9%; *p* < 0.05) and LDL-C (men and women: −9%; *p* < 0.05), with small to moderate effect sizes compared with the other two groups. In addition, there were statistically significant differences in triglycerides (TG) within sex and treatment groups. Specifically, men in the MEX group were the only group to reduce TG (−10%; *p* < 0.05), whereas women in the ST group exhibited a 7% increase after the 24-month follow-up (*p* < 0.05). Both changes showed small effect sizes.

## 4. Discussion

This study showed that, after a 24-month intervention, distinct sex-specific patterns in CRF and exercise adaptations were observed between older men and women undergoing statin therapy. Women receiving statin therapy alone exhibited a 4% reduction in CRF, whereas men did not show any significant change after the follow-up period. Both men and women in the combined treatment group (i.e., STMEX) also exhibited distinct responses after follow-up. Multicomponent exercise training reversed the decline in CRF in women in the combined treatment group, but not in men. Notably, statin therapy and/or age-related decline may have blunted the effects of exercise on CRF in men in the combined treatment group. These findings partially support our hypothesis of an interaction effect between statin therapy and exercise on CRF adaptations in women, but not in men.

The findings of the present study are consistent with those of previous experimental studies showing that statin therapy may interfere with exercise training benefits by impairing CRF [22,23,33]. For instance, Mikkus et al. demonstrated that simvastatin blunted the effects of a 12-week aerobic exercise training program in adults with metabolic syndrome compared with exercise training alone (ST + EX = 1.5% vs. EX = 10%) [23]. Furthermore, a cross-sectional study reported that men using statins had lower VO_2_peak values than women [34]. Notably, our study did not find any significant long-term changes in CRF among men receiving statin therapy alone. However, our results suggest an attenuated adaptation response to exercise training in men in the combined treatment group (STMEX = +1% vs. MEX = +21%). In contrast, women’s CRF decreased under statin therapy alone, but this effect appeared to be reversed by exercise training (STMEX = +27% vs. MEX = +27%). Despite the potential association between statin treatment and CRF adaptations observed in our study, contradictory evidence from observational studies also exists [18,19,20,21]. These divergent findings across studies may be related to: (1) methodological design; (2) participant characteristics; (3) exercise training protocols; (4) statin dosage and type; and (5) the potential influence of age-related decline, which cannot be excluded in our study.

In the present study, the differences observed between men and women may be explained by sex-specific variations in cardiovascular function (e.g., greater heart size, stroke volume, and cardiac output in men) [35], pulmonary adaptations, and age-related changes in body composition, skeletal muscle mass, hormonal profiles, and diet. Our functional status outcomes support these distinct sex-specific CRF adaptation patterns, as both sexes in the statin groups exhibited reductions in upper- and lower-limb strength. Physiologically, men generally have greater lean body mass, skeletal muscle strength, and muscle fiber cross-sectional area than women. Conversely, women tend to have greater fat mass deposition in the lower limbs [6,7,36]. However, aging alters body composition in both sexes by reducing lean body mass and increasing fat mass deposition [7]. In addition, men generally exhibit higher peak oxygen consumption because of enhanced respiratory gas exchange and reserve capacity compared with women [6,35], which may contribute to sex-specific differences in CRF responses to exercise training. Nevertheless, the exact mechanisms underlying these sex-specific CRF adaptation patterns remain unclear.

Based on previous studies [13,14,22,23,33], our hypothesis is that statins may impair cellular and molecular processes involved in mitochondrial bioenergetics, fat oxidation, and skeletal muscle biogenesis [13,14,22,23,33], thereby contributing to impaired skeletal muscle function and cardiovascular metabolism. However, we cannot exclude the independent effects of aging on CRF and functional status over the 24-month follow-up period, as no untreated control group was included. Therefore, further mechanistic studies are needed to better understand the effects of statins on skeletal muscle mass, muscle function, and exercise capacity responses in both sexes.

The magnitude of improvement observed in the exercise groups in both sexes was clinically relevant. Gains in CRF, assessed using the 6-min walk test, ranged from 17.3 to 21.6 m in men and women. Although a minimal clinically important difference for the 6-min walk test has not been specifically established for older adults with dyslipidemia, studies involving related cardiovascular populations suggest that improvements ranging from 14 to 50 m are associated with a reduced risk of major adverse cardiovascular events [37,38,39,40]. Likewise, each 1-MET increase in CRF has been associated with a 12% to 15% reduction in all-cause and cardiovascular mortality risk [41]. Therefore, the CRF improvements observed in the exercise groups (i.e., MEX and STMEX) in both sexes may indicate a substantial reduction in long-term cardiovascular risk, benefits that were not observed with statin therapy alone.

Regarding lipid metabolism, a 7% increase in triglyceride levels was observed in women in the statin group. This finding is consistent with a previous study in which statin therapy impaired CRF and reduced skeletal muscle mitochondrial density [23]. The same study also reported increases in lipid profile markers [23]. Lipid metabolism is regulated by distinct multi-tissue pathways linking triglycerides stored in white adipose tissue to mitochondrial function in organs such as skeletal muscle and the liver [37]. Thus, impaired mitochondrial bioenergetics may compromise beta-oxidation and consequently increase circulating and stored cholesterol and triglyceride levels [23,33], a pattern observed in women in the statin group. Despite the positive lipid-lowering effects of statins on total cholesterol (TC) and LDL-C levels (men: TC = −10% and LDL-C = −9%; women: TC = −9% and LDL-C = −9%) [38], both sexes receiving statin therapy exhibited a deterioration in functional status over time. Therefore, our findings suggest that long-term statin use may negatively affect functional markers related to skeletal muscle mass and strength, which, combined with the effects of aging, may further aggravate CRF and other age-related conditions independently of improvements in lipid metabolism.

This study is not without limitations, including: (i) the non-randomized design; (ii) the lack of a control group without exercise or statin therapy to assess the isolated effects of aging; (iii) unequal sample sizes across sex and treatment groups; and (iv) the inability to fully control for all potential confounding variables. Nevertheless, we attempted to mitigate these limitations by adopting statistical procedures designed to minimize their effects, including adjustment for important covariates such as age, number of comorbidities, and baseline values. In addition, intervention effect sizes within each sex and treatment group were calculated using Hedges’ g to account for differences in sample size. However, residual confounding due to unknown or incompletely measured factors cannot be excluded. Differences in statin dosage and the inability to monitor medication adherence also represent important limitations that may influence the interpretation of the observed outcomes. Therefore, these findings should be interpreted with caution.

## 5. Conclusions

The findings of the present study suggest distinct sex-specific patterns in CRF among older men and women with dyslipidemia undergoing statin therapy. Long-term statin monotherapy, together with aging, was associated with poorer CRF over time in women, but not in men. Regular multicomponent exercise training may reverse the decline in CRF observed in women. In men, long-term statin therapy was associated with blunted exercise training effects on CRF. Further research, particularly randomized controlled trials using different analytical approaches, is needed to confirm these findings.

## Figures and Tables

**Table 1:** Participants’ descriptive characteristics at baseline using a one-way analysis of variance to determine differences between gender and treatment, with Gabriel post hoc multiple comparisons correction adjustment.

Variables	Total (N = 981)	Male (N = 263)			Female (N = 718)		
Group, *n*		MEX (n = 76)	ST (n = 81)	STMEX (n = 106)	MEX (n = 222)	ST (n = 97)	STMEX (n = 399)
Age, years	66.9 ± 7.8	68.8 ± 6.5	66.3 ± 7.0	70.2 ± 7.3 *^†^	65.2 ± 8.2 *^‡^	62.3 ± 7.2	67.9 ± 7.5 **^⸹^
6-min walk test, m	445 ± 108	501 ± 130	455 ± 67	471 ± 113	441 ± 108	439 ± 67	429 ± 110
SBP, mmHg	139 ± 18	142 ± 15 *^‡^	134 ± 15	143 ± 16 *^†^	140 ± 18 **^‡^	129 ± 17	141 ± 18 **^†^
DBP, mmHg	79 ± 11	81 ± 10	77 ± 8	81 ± 13 *^†^	79 ± 12 *^‡^	75 ± 9	79 ± 11 *^†^
Total cholesterol, mg/dL	202 ± 39	194 ± 35	192 ± 38	180 ± 38 *^⸹^	209 ± 34	208 ± 39	205 ± 41
HDL cholesterol, mg/dL	53 ± 14	47 ± 11	46 ± 12	50 ± 11	54 ± 12	55 ± 13	56 ± 17
LDL cholesterol, mg/dL	124 ± 34	125 ± 32	118 ± 31	105 ± 34 *^⸹^	134 ± 27	126 ± 34	123 ± 36 *^⸹^
Triglycerides, mg/dL	124 ± 58	116 ± 53	136 ± 78	125 ± 61	112 ± 45	122 ± 62	131 ± 61 **^⸹^
Glycaemia, mg/dL	105 ± 39	103 ± 24	118 ± 124	110 ± 30	98 ± 19 *^‡^	107 ± 27	106 ± 29 *^⸹^
HbA1c, %	6.7 ± 1.1	6.4 ± 0.9	6.5 ± 1.4	6.6 ± 0.9	6.4 ± 1.1	6.8 ± 1.1	6.8 ± 1.1
Body mass, kg	74.0 ± 12.0	77.5 ± 10.8	81.7 ± 12.1	82.4 ± 12.8	72.0 ± 10.5	72.3 ± 10.3	71.0 ± 11.0
Waist circumference, cm	91.5 ± 9.7	94.1 ± 7.0	96.3 ± 8.7	99.4 ± 8.9 **^⸹^	89.4 ± 9.2	87.0 ± 9.6	90.4 ± 9.3 *^†^
Body mass index, kg/m^2^	28.9 ± 4.1	27.5 ± 3.5	28.3 ± 3.6	29.5 ± 4.6 *^⸹^	28.9 ± 4.1	28.8 ± 4.0	29.1 ± 4.1
Waist-to-hip ratio	0.90 ± 0.07	0.95 ± 0.05	0.97 ± 0.07	0.98 ± 0.05 *^⸹^	0.86 ± 0.06	0.87 ± 0.07	0.88 ± 0.06 *^⸹^
Comorbidity, n	2.4 ± 1.6	1.8 ± 1.3 *^‡^	2.4 ± 1.4	2.1 ± 1.4	2.3 ± 1.5	2.5 ± 1.6	2.5 ± 1.7
Hypertension, n (%)	489 (50)	28 (10)	39 (15)	52 (19)	113 (15)	44 (6)	213 (27)
Type II Diabetes, n (%)	199 (20)	17 (6)	18 (7)	37 (14)	19 (2)	13 (2)	95 (12) ^†⸹^
Osteoarthrosis, n (%)	284 (29)	8 (3)	13 (5)	18 (7)	76 (10)	28 (4)	141 (18)
Osteoporosis, n (%)	233 (24)	4 (2)	1 (1)	6 (2)	70 (9)	27 (4)	125 (16)

Data are expressed as mean ± SD. DBP: Diastolic blood pressure. HDL: High-density lipoprotein. LDL: Low-density lipoprotein. MEX: Multicomponent exercise training. SBP: Systolic blood pressure. ST: Statins. STMEX: Statins combined with multicomponent exercise training. * Differences between evaluations (*p* ≤ 0.05). ** Differences between evaluations (*p* ≤ 0.001). ^†^ Differences between STMEX vs. ST. ^‡^ Differences between MEX vs. ST. ^⸹^ Differences between STMEX vs. MEX.

## Data Availability

The data described in this manuscript will be made available upon reasonable request pending approval by the corresponding author due to privacy and confidentiality restrictions.

## Supplementary Materials

The following supporting information can be downloaded at Figshare public access data repository https://doi.org/10.6084/m9.figshare.22634758 (accessed on 14 May 2026), Supplemental Table S1: Comparison between groups in functional status and lipid profile after the 24-month intervention; Supplemental Table S2: Differences between groups in functional status and lipid profile after 24-month intervention; Supplemental Table S3: Percentage differences and Hedges’ g effect size within groups in functional status and lipid profile from baseline to 24-month intervention.

## Abbreviations

The following abbreviations are used in this manuscript:

1-RM	One-repetition maximum
BM	Body mass
BMI	Body mass index
CRF	Cardiorespiratory fitness
CVD	Cardiovascular disease
HbA1c-	Hemoglobin glycated
HDL-C	High-density lipoprotein cholesterol
HMG-CoA	hydroxymethylglutaryl-coenzyme A
HR max	Heart rate maximum
LDL-C	Low-density lipoprotein cholesterol
LLS	Lower-limb strength
MET	Metabolic equivalent of task
MEX	Multicomponent exercise training
ST	Statins
STMEX	Statins combined with multicomponent exercise training
TC	Total cholesterol
TG	Triglycerides
ULS	Upper-limb strength
VAB	Velocity, agility and dynamic balance
VO2max	Volume oxygen consumption maximum
WC	Waist circumference
WHR	Waist-to-hip ratio
